# Genetically Predicted Circulating Concentrations of Micronutrients and COVID-19 Susceptibility and Severity: A Mendelian Randomization Study

**DOI:** 10.3389/fnut.2022.842315

**Published:** 2022-04-25

**Authors:** Neil Daniel, Emmanouil Bouras, Konstantinos K. Tsilidis, David J. Hughes

**Affiliations:** ^1^Cancer Biology and Therapeutics Laboratory, School of Biomedical and Biomolecular Sciences, Conway Institute, University College Dublin, Dublin, Ireland; ^2^Department of Hygiene and Epidemiology, University of Ioannina School of Medicine, Ioannina, Greece; ^3^Department of Epidemiology and Biostatistics, School of Public Health, Imperial College London, London, United Kingdom

**Keywords:** Mendelian randomization, GWAS, micronutrients, supplements, SARS CoV-2, COVID-19

## Abstract

**Background:**

Coronavirus disease 2019 (COVID-19) is caused by the severe acute respiratory syndrome coronavirus 2 (SARS-CoV-2) which since 2019 has caused over 5 million deaths to date. The pathogenicity of the virus is highly variable ranging from asymptomatic to fatal. Evidence from experimental and observational studies suggests that circulating micronutrients may affect COVID-19 outcomes.

**Objectives:**

To complement and inform observational studies, we investigated the associations of genetically predicted concentrations of 12 micronutrients (β-carotene, calcium, copper, folate, iron, magnesium, phosphorus, selenium, vitamin B-6, vitamin B-12, vitamin D, and zinc) with SARS-CoV-2 infection risk and COVID-19 severity using Mendelian randomization (MR).

**Methods:**

Two-sample MR was conducted using 87,870 individuals of European descent with a COVID-19 diagnosis and 2,210,804 controls from the COVID-19 host genetics initiative. Inverse variance-weighted MR analyses were performed with sensitivity analyses to assess the impact of potential violations of MR assumptions.

**Results:**

Compared to the general population, nominally significant associations were noted for higher genetically predicted vitamin B-6 (Odds ratio per standard deviation [*OR*_SD_]: 1.06; 95% confidence interval [CI]: 1.00, 1.13; *p*-value = 0.036) and lower magnesium concentrations (*OR*_SD_: 0.33; 95%CI: 0.11, 0.96; *P* = 0.042) with COVID-19 infection risk. However, the association for magnesium was not consistent in some sensitivity analyses, and sensitivity analyses could not be performed for vitamin B-6 as only two genetic instruments were available. Genetically predicted levels of calcium, folate, β-carotene, copper, iron, vitamin B-12, vitamin D, selenium, phosphorus, or zinc were not associated with the outcomes from COVID-19 disease.

**Conclusion:**

These results, though based only on genetically predicated circulating micronutrient concentrations, provide scant evidence for possible associations of micronutrients with COVID-19 outcomes.

## Introduction

Coronavirus disease 2019 (COVID-19) is caused by the severe acute respiratory syndrome coronavirus 2 (SARS-CoV-2). Since the beginning of the pandemic, there have been over 275,233,892 confirmed cases and over 5,364,966 deaths attributed to COVID-19 (1). The pathogenicity of the virus is highly variable ranging from asymptomatic to fatal. In severe cases, an immune system hyper-reaction coupled with a marked cytokine and chemokine release (“cytokine storm,” hypercytokinemia) has been observed. This severely damages the lung epithelium, exacerbates secondary microbial infections, and affects other organ systems, which can result in septic shock, multi-organ failure, and death (2). Factors associated with COVID-19 outcomes include age, body mass index (BMI), physical activity levels, and pre-existing conditions (3–6). To date, the antiviral drugs Veklury (remdesivir) and Paxlovid (nirmatrelvir and ritonavir) and the monoclonal antibodies casirivimab and imdevimab are the approved treatments by the US Federal Drug Administration (FDA). The European Medicines Agency (EMA) has approved four vaccines for use: The Jansenn and AstraZeneca vaccines utilize non replicating viral vectors while the Pfizer and Moderna vaccines use newer mRNA technology (7). The FDA is yet to approve the AstraZeneca vaccine.

Previously, various micronutrients have been identified as essential for immunocompetence, particularly folate, iron, selenium, zinc, and vitamins A, C, D, E, B2, B6, and B12 (8). By extension, micronutrient deficiencies are recognized as a global public health hurdle as poor nutritional status predisposes many populations to infection (9).

Observational and ecologic studies have proposed a link between the levels of micronutrients such as vitamin D, selenium, and zinc with COVID-19 disease severity (10–12). However, observational studies are vulnerable to confounding and reverse causation bias. Mendelian randomisation (MR) is a genetic epidemiology technique that uses genetic variants as variables to determine the potential causal effect of an exposure, such as micronutrient level (13). As genetic alleles are randomly distributed throughout the population, associations with environmental confounding variables are negated. Similarly, since genetic alleles are present before the development of COVID-19, they cannot be affected by reverse causation.

Genetic variants associated with circulating vitamin D concentrations have been linked to COVID-19, implicating low levels of vitamin D in severe disease (14). Conversely, genetic determinants of serum 25-hydroxyvitamin D3 (calcifediol) levels, gleaned from a genome-wide association study (GWAS), and meta-analysis of 443,734 participants were used to determine the link between elevated calcifediol levels and COVID-19 susceptibility and severity. The authors concluded that there was insufficient evidence to support an association between calcifediol levels and COVID-19 susceptibility, severity, or hospitalization. They recommended against vitamin D supplementation use to improve COVID-19 outcomes (15). Similar findings were reported from multiple MR studies which found no associations between genetically predicted circulating vitamin D levels and COVID-19 infection and severity (16–19).

Calcium signaling is indispensable in immunity, and hypocalcemia is frequently observed in critically ill patients (20, 21). In cases of COVID-19, lower serum calcium has been associated with increased clinical severity and less favourable prognosis (22, 23).

Copper has been identified as essential for proper immune functioning by the European Food Safety Authority (24). It is a free radical scavenger and is also required for the appropriate functioning of T helper cells, B cells, neutrophils, natural killer (NK) cells, and macrophages with deficiencies linked to impaired immunity (25, 26). Further, copper levels are seen to be locally elevated following infection of the lungs (27).

Folate deficiency is commonly observed in individuals susceptible to infection (28). The number of circulating T cells, B cells, and NK cells found in folate deficient rats is significantly lower than in controls (29). *In vitro*, folate deficiency inhibits the proliferation of human primary CD8+ T cells (30). *In vivo*, low folate status has also been associated with lower levels of proteins involved in the activation and regulation of immune function (31).

Iron is an indispensable component of multiple enzymes involved in immune cell activity. Deficiencies are therefore associated with reduced efficacy of lymphocytes, NK cells and cytokine signalling (24, 32). The trace element selenium and proteins containing the modified amino acid selenocysteine (in which selenium is incorporated into selenoproteins) have roles in the proper functioning of both the innate and adaptive immune systems (33). Selenium also acts to alleviate oxidative stress, an emerging characteristic of COVID-19 (34).

Zinc deficiency has been linked to immune dysfunction and the resultant vulnerability to infection. Loss of zinc homeostasis results in the abnormal formation of lymphocytes, impaired NK cell activity, altered cytokine signaling, and reduced phagocytosis (35). Zinc deficiency is also linked to impaired T-cell mediated antibody response and abnormal complement activity (36). Furthermore, zinc has been identified as a critical factor in anti-viral immunity and is currently being extensively investigated in randomized clinical trials as a component of treatment regimens aimed at improving COVID-19 outcomes (37, 38).

Levels of phosphorus have been observed to be inversely correlated with COVID-19 clinical severity, while COVID-19 patients with low phosphorus are more likely to have altered lung function and require admission to the intensive care unit (ICU) (39).

Following potassium, magnesium is the second most abundant intracellular cation and is essential in over 600 enzymatic reactions including those contributing to the exaggerated immune and inflammatory responses characteristic of COVID-19 pathogenesis (40). A growing body of evidence suggests that magnesium protects against respiratory system diseases and may have a role in the prevention of COVID-19 (41).

Vitamin B-6 has long been recognized as having pivotal roles in the maintenance of lymphocyte development, NK cell activity, and immunoglobin production (42, 43). Similarly, vitamin B-12 is also essential for lymphocyte expansion and NK cell activity. Lower B-12 levels are linked to lower concentrations of circulating lymphocytes and accompanying immunity (44).

Finally, a meta-analysis of prospective studies has identified dietary and circulating β-carotene as inversely associated with the risk of all-cause mortality (45). This may be due to its multifunctional roles in health including its free radical scavenging and pro-vitamin A activity which may bolster both adaptive and innate immune function (46, 47).

Several of these micronutrients have been suggested for supplement use to mitigate COVID-19 disease, such as selenium and zinc (48), but the evidence underpinning this is mainly theoretical or based on small COVID-19 patient-cohort studies. Here, we have utilized alleles as proxies for the genetically predicted circulating status of β-carotene, calcium, copper, folate, iron, magnesium, phosphorus, selenium, zinc, and vitamins B-6 and B-12 to assess the association by MR with the development and severity of COVID-19 in a large and multi-cohort dataset.

## Methods

### Data for the Genetic Epidemiology of COVID-19

We used the European-specific summary genetic association estimates from the COVID-19 host genetics initiative^1^ to test the association of genetically predicted micronutrient levels for four COVID-19 outcomes listed below. We used the GWAS Release 5 for comparisons (i) + (ii) and Release 6 for (iii) + (iv):

**(i) Severe COVID-19**, defined as hospitalization with laboratory confirmed SARS-CoV-2 infection as the primary reason for the admission followed by death or respiratory support vs. population controls (cases = 4,606, controls = 702,801),
**(ii) Case-only COVID-19 hospitalization**, defined as hospitalization with laboratory confirmed SARSCoV-2 infection due to COVID-19-related symptoms vs. non-hospitalization SARS-CoV-2 infection due to COVID-19-related symptoms (cases = 4,829, controls = 11,816),
**(iii) COVID-19 hospitalization**, defined as hospitalization with laboratory confirmed SARS-CoV-2 infection due to COVID-19-related symptoms vs. population controls (cases = 17,992, controls = 1,810,493), and
**(iv) COVID-19 infection**, defined as laboratory confirmed SARS-CoV-2 infection (RNA and/or serology based), physician diagnosis of COVID-19, or self-report as COVID-19 positive vs. population controls (cases = 87,870, controls = 2,210,804).

Details on data collection and assessment are provided in Supplementary Table 1.

### Data for the Genetic Epidemiology of Circulating Micronutrient Concentrations

Details on the identification of genetic instruments for the 12 micronutrients are described elsewhere (49). In brief, we performed a search of published GWASs conducted among individuals of European descent on circulating concentrations of minerals and vitamins in the GWAS catalog and PubMed (last search performed in October 2019). After excluding micronutrients for which no genome-wide significant results have been reported, or summary genetic association estimates were adjusted for BMI, we identified 12 micronutrients (namely β-carotene, calcium, copper, folate, iron, magnesium, phosphorus, selenium, vitamins B6, B12, and D, and zinc) that may be associated with COVID-19 outcomes (50–60). The selected GWASs included data from 12 European countries and the United States. The participants were predominantly female, making up from 55 to 69% of the total sample size. Two individual GWASs were used to instrument calcium concentrations (59, 60). Single nucleotide polymorphisms (SNPs) with minor allele frequency (MAF) of <5% in the GWASs were omitted as their association estimates with the micronutrients may be inaccurate. SNPs that were associated with the circulating concentrations of the micronutrients at a genome-wide significance level (*p* < 5 × 10^−8^) and were not in linkage disequilibrium (linkage disequilibrium *r*^2^ ≤ 0.01) were included. This yielded summary genetic association data for 333 common (MAF ≥ 0.05) SNPs that were robustly associated with the 12 micronutrient concentrations (Supplementary Table 2).

### Statistical Power

Power calculations were performed using an online tool available at http://cnsgenomics.com/shiny/mRnd/ (61). The statistical power to capture an odds ratio (*OR*) of 1.10 or 0.9 per standard deviation (*SD*) change in the genetically predicted circulating concentrations ranged from 0.08 (folate) to 0.31 (copper) for risk of severe COVID-19, from 0.07 (folate) to 0.23 (copper) for risk of COVID-19 hospitalization among COVID-19 cases, from 0.16 (folate) to 0.92 (zinc) for risk of COVID-19 hospitalization, and from 0.07 (folate) to 1.00 (zinc) for risk of COVID-19 infection. The statistical power was >0.80 for copper, calcium, and zinc regarding the risk of COVID-19 hospitalization and for all the instruments tested except phosphorus and folate concerning the risk of COVID-19 infection (Supplementary Table 3). To ensure the robustness of all findings, all associations with <50% power were excluded from the analysis. This excluded all instruments for assessing the risk of severe COVID-19 *vs*. the population and the risk of COVID-19 hospitalization among COVID-19 cases (outcomes I and II). All nutrients were above this threshold for COVID-19 infection *vs* the population while β-carotene, selenium, iron, copper, calcium, zinc and vitamins B6, B12 and D remained for hospitalization due to COVID-19 *vs* the population (outcomes iii and iv) (Supplementary Table 3). Thus, only outcomes iii and iv were further analyzed and presented here.

### Mendelian Randomization Analysis

A two sample MR using summary association data from GWASs of circulating micronutrients and COVID-19 outcome was performed. The genetically predicted micronutrient concentrations associations with COVID-19 *vs* the population and hospitalized COVID-19 *vs* the population were assessed. This analysis used genomic data yielded from the COVID-19 Host Genetics Initiative which combined genetic data from 87,870 cases and two million controls across 46 distinct studies (62). A single SNP was available for β-carotene, the effect estimate of which was calculated as the ratio of the SNP-outcome divided by the SNP–nutrient association (63). For instruments composed of multiple SNPs, the random-effects inverse variance-weighted (IVW) method was employed. The IVW analysis is comparable to a meta-analysis of single SNP effects (64). The β estimates and standard errors (SEs) from the regressions for circulating concentrations of β-carotene, copper, selenium, vitamin B-6, and zinc were transformed from the logarithmic scale provided in the published GWAS to the natural scale using an available formula (65). All reported associations correspond to an OR for COVID-19 outcome per *SD* change in the genetically predicted circulating concentrations of the nutrients. *P* values of <0.05 were deemed significant and high-confidence was assigned to findings that survived multiple-testing adjustment with a False Discovery Rate of 5%. All analyses were prespecified and performed using R Core Team (2020) version 3.4.3, R Foundation for Statistical Computing, Vienna, Austria, using the MendelianRandomization package.

### Methods to Assess the Robustness of MR Findings

**Figure 1:**
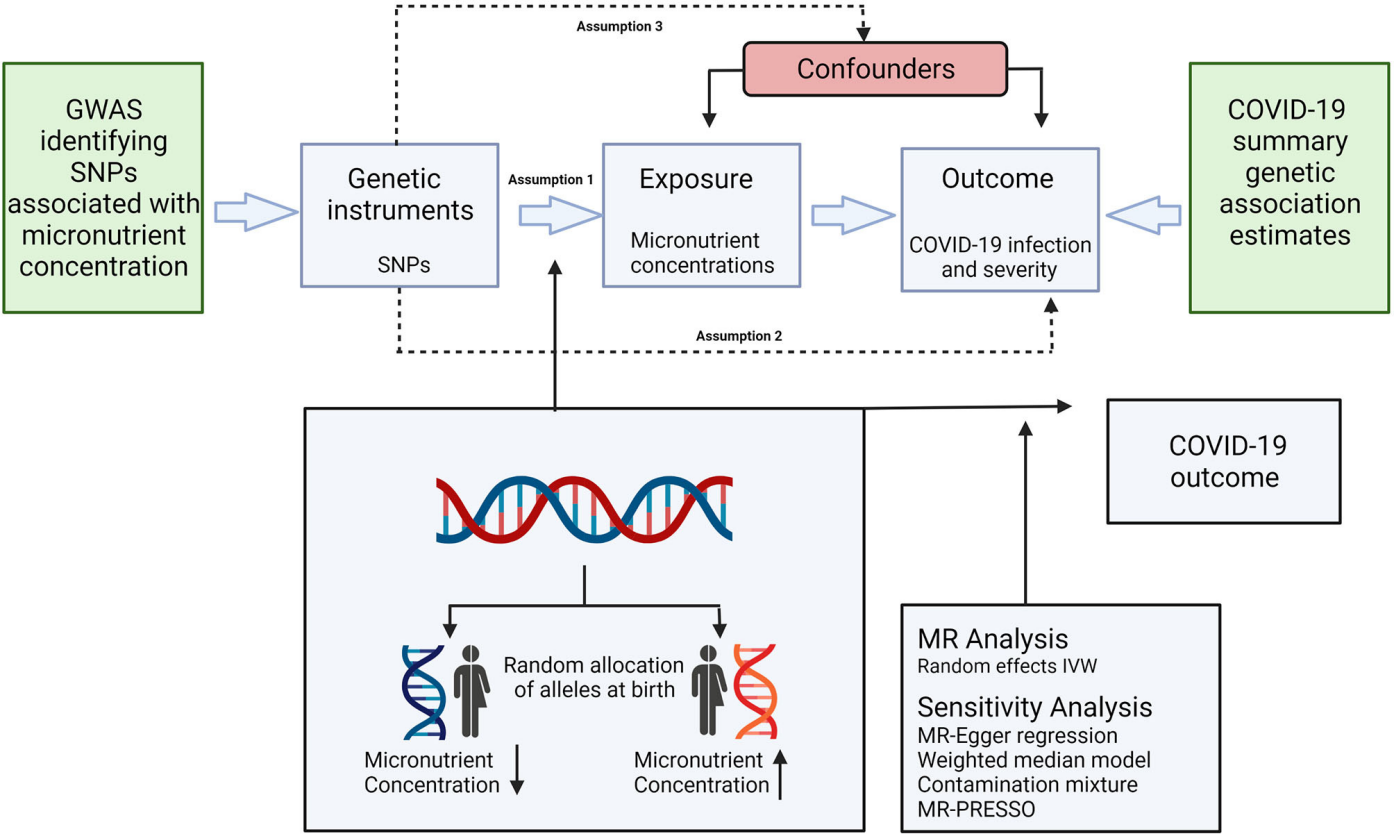
Schematic representation of the Mendelian randomization assumptions and study design. Genetic instruments are selected based on their association with the concentration of a specific micronutrient (assumption 1), but do not directly impact the assessed outcomes (assumption 2), or any confounders of micronutrient concentrations and the assessed outcomes (assumption 3). IVW, inverse variance weighted; SNP, single nucleotide polymorphism; GWAS, genome-wide association study; MR, Mendelian randomization; MR-PRESSO, Mendelian randomization pleiotropy residual sum and outlier.

Mendelian Randomisation analysis is dependent on assumptions as illustrated in Figure 1. It is a requisite of the IVW method that all genetic instruments are associated with the concentration of their relevant micronutrient (relevance assumption), but do not directly impact the assessed outcomes (exclusion restriction), or any confounders of micronutrient concentrations and the assessed outcomes (independence assumption) (66). The strength of the genetic instruments was assessed using the *F* statistic formula: *F* = R2 (*n* – 2)/(1 – R2), where R2 is the proportion of the variance of the micronutrient concentration explained by each genetic instrument and *n* is the sample size of the GWAS (67). The *F* statistics in the current study ranged from 36 to 420 for all genetic instruments, indicating an absence of weak instruments as all values were >10 (63).

Horizontal pleiotropy can potentially cause violations of the exclusion restriction assumption (genetic instruments being independent of the outcome, conditional on the risk factor, and confounders) and was investigated using descriptive and statistical analyses. The Cochran's *Q* statistical test for heterogeneity was utilized to determine the extent to which any differences in the individual effect sizes among each of the selected genetic instruments may be different (e.g., due to pleiotropy) (68). Low heterogeneity across the SNP instruments would be expected for valid instruments resulting in high Q *p*-values for the statistical tests and metrics of heterogeneity. We further evaluated whether the selected genetic instruments were associated with secondary phenotypes in the PhenoScanner database.^2^

Where the number of genetic instruments was ≥3, MR-Egger regression, weighted median, and contamination mixture methods were performed. MR-Egger can detect horizontal pleiotropy and provide an effect estimate which is not subject to this violation of the standard instrumental variable assumption. The intercept from MR-Egger regression indicates potential pleiotropy, a low *p*-value suggests either pleiotropy or failure of the InSIDE (INstrument Strength Independent of Direct Effect) assumption, whereas the slope can be interpreted as the circulating nutrient effect on COVID-19 outcome adjusted for horizontal pleiotropy (70). The weighted median estimator is used to combine data on multiple genetic variants into a single causal estimate. This estimate remains valid even when up to 50% of the instrumental variables are invalid (71). The contamination mixture employs a likelihood-based approach using the variant-specific causal estimates, and under the assumption that there is a single causal effect of the risk factor on the outcome, it can estimate this effect robustly and efficiently, even in the presence of some invalid genetic variants (72). When the number of genetic instruments ≥4, MR-PRESSO was also employed to identify SNP outliers, and analyses were rerun after omitting these variants (73).

## Results

**Figure 2:**
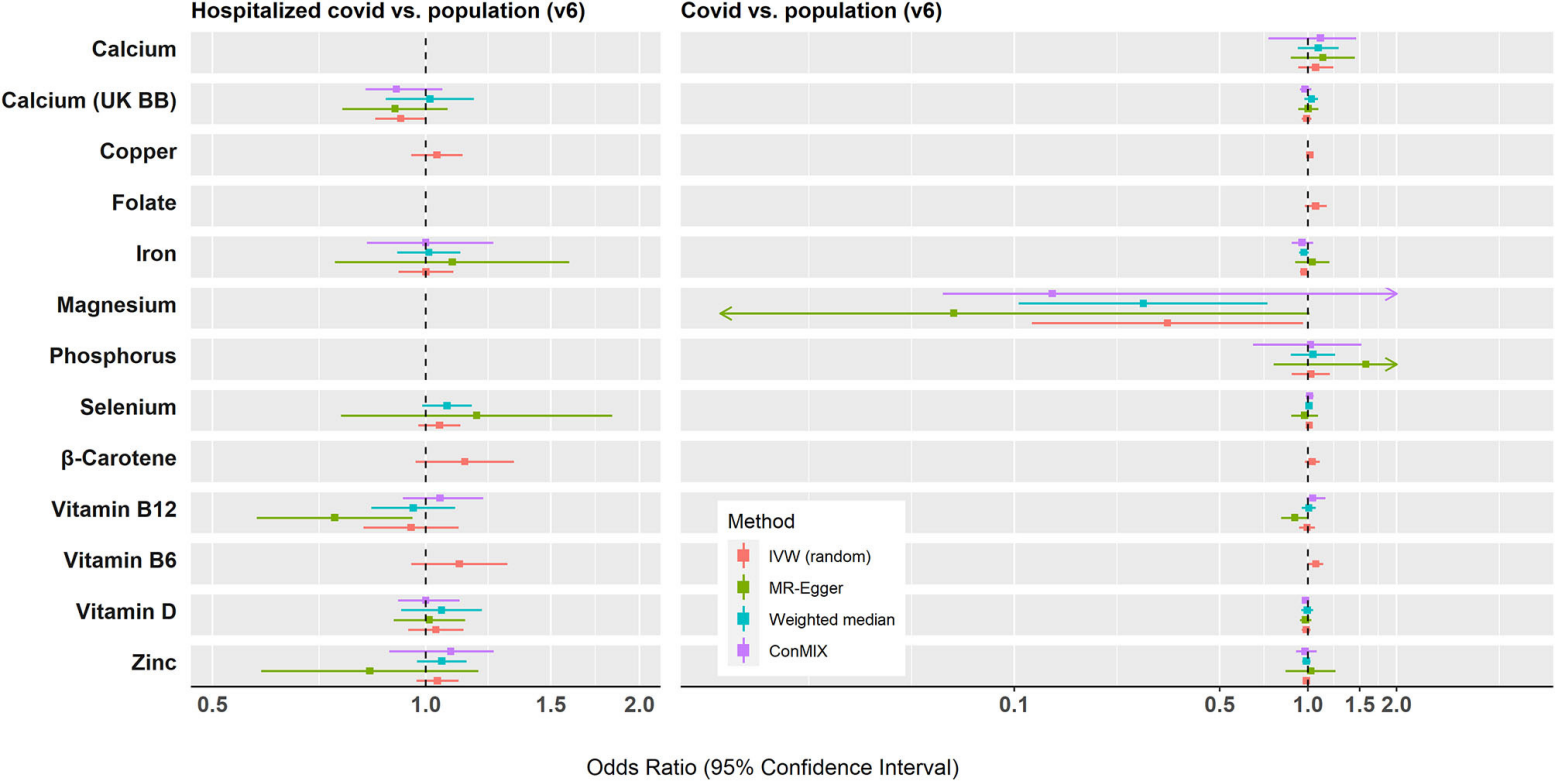
Summary of the Mendelian randomization results for the associations between genetically predicted micronutrient concentrations and COVID-19 outcomes. IVW, inverse variance weighted; conMIX, contamination mixture.

Genetically predicted magnesium concentrations were lower while predicted vitamin B6 concentrations were higher in the COVID-19 cases relative to the population. No significant associations were found between the assessed COVID-19 outcomes and genetically predicted levels of folate, calcium, copper, iron, phosphorus, selenium, zinc, β-carotene, or vitamins D and B12. All associations using the IVW models and MR sensitivity analysis methods are shown in Figure 2. Supplementary Table 4 shows associations using the MR sensitivity analyses methods for each of the COVID-19 outcomes studied.

A nominally significant association was observed for each one *SD* (0.08 mmol/L) decrease in genetically predicted magnesium concentration and risk of COVID-19 infection relative to the population (*OR*_SD_: 0.33, 95% *CI*: 0.11, 0.96; *p* = 0.04, *n* = 5 SNPs). There was some evidence for heterogeneity in the association (*I*^2^ = 68%, Cochran's *Q* test *p* = 0.015). This association did not survive correction for multiple testing (FDR *q*-value = 0.29). There was little evidence of horizontal pleiotropy based on the MR-Egger intercept test (*P* = 0.208). The weighted median method yielded results similar to the IVW (*OR*: 0.27; 95% *CI*: 0.10, 0.73; *p* = 0.009), while the contamination mixture produced consistent results but with very wide *CI*s (*OR*: 0.13; 95% *CI*: 0.06, 3.73; *p* = 0.34). The MR PRESSO analysis did not reveal any outlying SNPs at α = 0.05. However, several of the genetic instruments for magnesium have been associated in GWASs with phenotypes (e.g., BMI, kidney function, and blood pressure) that may indicate horizontal pleiotropy in relation to COVID-19 (Supplementary Table 5). When rs4072037, which has been previously associated with BMI, was removed from the analysis, the resulting IVW estimate was attenuated (*OR*_SD_: 0.84, 95% *CI*: 0.37, 1.94; *I*^2^ = 0%) (74). Little evidence was found supporting the hypothesis that magnesium concentrations differ between hospitalized COVID-19 cases and the general population.

Genetically predicted concentrations of vitamin B-6 were found to be higher in COVID-19 cases relative to the population (*OR*_SD_: 1.06; 95% *CI*: 1.00, 1.13; *p* = 0.036). This association did not retain significance following correction for multiple testing (*q* = 0.29). As only two instruments were used, MR-Egger regression, weighted median, and contamination mixture methods were not carried out. These instruments (rs4654748 and rs1256335) were not associated in GWASs with phenotypes that may indicate horizontal pleiotropy in relation to COVID-19. There was no association between predicted vitamin B-6 concentrations and hospitalized COVID-19 *vs*. population.

## Discussion

In this multi-cohort MR analysis of 12 circulating micronutrient concentrations and COVID-19 infection and outcome, we observed that genetically predicted levels of circulating magnesium in COVID-19 cases were lower relative to the population. Surprisingly, we found evidence that genetically predicted levels of vitamin B6 were higher in COVID-19 patients relative to the population. These associations did not survive correction for multiple testing and sensitivity MR analyses were either not fully consistent (for magnesium) or not performed (for vitamin B6). We observed little evidence that genetically predicted circulating concentrations of any of the other examined micronutrients (i.e., folate, calcium, zinc, selenium, copper, iron, phosphorus, β-carotene and vitamins B12 and D concentrations) were associated with either assessed COVID-19 outcome.

### Magnesium and COVID-19 Outcome

The present study observed lower levels of genetically predicted circulating magnesium in COVID-19 cases relative to the population. This trend of lower magnesium was also observed for both assessed COVID-19 outcomes, although only COVID-19 infection *vs*. population was nominally significant. Magnesium (Mg^2+^) has previously been associated with anti-viral immunity as mutations of the magnesium transporter 1 (*MAGT1*) gene causes an immunodeficiency called X-linked immunodeficiency with Mg^2+^ defect, EBV infection, and neoplasia (XMEN) which is characterized by CD4 lymphopenia, chronic viral infections, and impaired T lymphocyte activation (75). Lower plasma Mg^2+^ has also been associated with higher EBV viral load in non-XMEN women (76). Mechanistically, reduced intracellular free Mg^2+^ causes diminished expression of NKG2D, the NK activating receptor in NK and CD8+ T cells, thereby reducing their cytolytic activity. Interestingly, magnesium supplementation restores intracellular free Mg^2+^ and NKG2D expression in patients with XMEN and reduced EBV-infected cells *in vivo* (77). Low levels of intracellular free Mg^2+^ also lead to increased expression of the immune checkpoint programmed cell death 1 (PD-1) along with lower expression of NKG2D in hepatitis B-infected NK and CD8+ T cells (78). This phenomenon has recently been observed in the NK cells of COVID-19 patients as they were seen to have increased expression of PD-1 and reduced expression of NKG2D relative to non-infected controls (79). It's possible that lower circulating magnesium contributes to a diminished immune response to COVID-19 by reducing the expression of NKG2D and increasing that of PD-1. There was some evidence of heterogeneity which may imply violation of one of the MR assumptions. Although the results of the weighted median approach were consistent with the IVW analysis, there was indication of horizontal pleiotropy violating the MR assumptions as several of the genetic instruments for magnesium have been associated with phenotypes (e.g., BMI, kidney function, blood pressure) that are also associated with COVID-19 outcomes. Together, these results cast doubt in the role of magnesium in COVID-19 disease trajectories.

### Vitamin B6 and COVID-19 Outcome

A potential protective role of vitamin B6 in ameliorating the impact of COVID-19 has been postulated based on its role in proper immune functioning and in suppression of major disease-associated processes such as inflammation, oxidative stress, and regulation of Ca^2+^ influx (80). However, we found a nominally significant association between higher levels of genetically predicted circulating vitamin B6 in those infected with COVID-19 relative to the population. Only two instrumental variables were available for our analysis therefore sensitivity MR analyses could not be run. Furthermore, we are not aware of any plausible biological mechanism whereby higher vitamin B6 would predispose individuals to COVID-19.

### Other Micronutrients and COVID-19 Outcomes

No associations were found between genetically predicted circulating calcium, folate, zinc, selenium, copper, iron, phosphorus, β-Carotene, or vitamin D and B12 concentrations, and COVID-19 outcome, contrasting with preliminary observational studies for some of these micronutrients (11, 32, 48, 81). Genetic instruments obtained from two separate GWASs were used to assess the associations of COVID-19 infection and hospitalization with predicted circulating calcium concentrations [up to 212 variants from the UK biobank analysis and 6 from a separate GWAS meta-analysis of European studies (59, 60)]. For each of these GWAS datasets, no association was observed for either outcome. These findings conflict with the existing literature as lower calcium has been found to be prevalent in hospitalized COVID-19 patients and concentrations have been inversely correlated with severity (82–84). It is possible that we did not find an association between genetically predicted low calcium and the assessed COVID-19 outcomes because the genetic instruments are not associated with the hypocalcemic levels (<2.20 mmol/L) observed in some patients with severe COVID-19 (82, 83).

Folate is an essential water-soluble micronutrient. Recently, a study proposed that pregnant women are 10-fold less likely to be hospitalized for a SARS-CoV-2 infection than for the 2009 H1N1 influenza pandemic, leading to speculation this may be due to folic acid supplements regularly taken by pregnant women (85). Here, in concordance with another recent study, genetically predicted folate concentrations were not associated with COVID-19 infection compared to the population (86).

Previously, serum copper levels in combination with age and selenoprotein P (SELENOP; the major plasma seleniumcontaining selenoprotein) concentration was applied to receiver operating characteristic (ROC) curve analysis to predict survival from COVID-19, yielding an area under the curve (AUC) of 95.0% (81). A similar analysis identified SELENOP and zinc in tandem with age as a composite biomarker and accurate predictor of COVID-19 survival by ROC analysis, yielding an AUC of 94.42% (48). Additionally, nutritive adjuvant therapy with selenium, zinc, and vitamin D has been recommended for high-risk groups (such as the elderly and those with low nutrient status) soon after the time of suspected infection with SARS-CoV-2 (87). These associations between zinc, selenium, and copper, and COVID-19 outcomes were not observed in the present study, instead, our findings replicate those of a more modestly sized MR study which assessed associations between genetically predicted concentrations of circulating zinc, selenium, copper (and vitamin K1) with the risk of infection, hospitalization, and critical illness due to COVID-19 (88). Our results also align with those of a study assessing associations between SNPs linked to selenium and zinc concentrations and COVID-19 severity (14).

Iron concentration has been reported to be inversely correlated with the severity of COVID-19 symptoms; however, excess serum iron has also been linked with increased inflammation and tissue fibrosis (89, 90). We found no evidence for an association between genetically predicted iron concentration and the assessed COVID-19 outcomes. Circulating levels of phosphorus are not typically associated with immunity; however, abnormal phosphorus serum levels have been associated with increased mortality levels due to community-acquired pneumonia (91). Additionally, low phosphorus levels were seen to be more prevalent in serum of severe COVID-19 patients than those with moderate disease (39). We found no association between genetically predicted circulating concentrations of phosphorus and COVID-19 infection. It is generally held that in addition to its pro-vitamin A activity, β-carotene, found in orange fruits and vegetables and some leafy green vegetables, may prevent oxidative damage (92, 93). Although initial studies suggested β-carotene supplementation may bolster health, later investigations found no beneficial effect in well-nourished people (94–97). Our study found no association between genetically predicted concentrations of β-carotene and risk of infection with COVID-19 or severity. Vitamin B12 can affect host immune responses to viral infections as well as inflammatory activity and circulating levels have been linked to clinical outcomes in COVID-19 patients (98, 99). These associations were not replicated in the present study. Finally, in agreement with multiple smaller MR studies, with COVID-19 case numbers ranging from 1,746 to 38,984, we found no associations between genetically predicted levels of circulating vitamin D and the assessed COVID-19 outcomes (16–19).

The discordance of our results with some previous studies may be due to several reasons, including the vastly larger sample size in the current study, and/or other dietary factors significantly affecting micronutrient levels in these COVID-19 patients, or reverse causality issues from low micronutrient levels in patients severely affected by COVID-19. Conversely, it may be that the micronutrient concentrations are not accurately predicted by the genetic instruments. This is perhaps especially relevant for markedly low or high micronutrient concentrations that may also be most important in patients vulnerable to COVID-19 fatality, as has been observed for measurements of selenium and zinc, although in a small patient cohort study (48).

### Strengths and Limitations

Biases present in traditional observational studies can be avoided by MR studies, but they are not without limitations. Here, we used summary level data which allowed us to incorporate large data sets, however, summarized data do not allow for stratification by factors such as sex, age, adiposity, diet, vitamin, and micronutrient supplement use, and co-morbidities. Furthermore, several micronutrients had few genetic instruments limiting statistical power for COVID-19 severity outcomes and the ability to perform sensitivity MR analyses. Finally, the SNPs used in our analysis predict circulating concentrations of the assessed micronutrients which may differ from those found in more clinically pertinent tissues, or those subjects most susceptible to fatality from COVID-19.

In conclusion, using a comprehensive MR study, we found scant evidence for possible associations of genetically predicted circulating concentrations of micronutrients with COVID-19 outcomes. The observed associations for magnesium (inverse) and vitamin B6 (positive) with the risk of COVID-19 infection should be interpreted with caution given the possibility for horizontal pleiotropy. Although the present study implemented data from 87,870 COVID-19 cases, it is possible our analysis did not capture the extremely low micronutrient concentrations that have previously been directly measured in critically ill COVID-19 patients. Furthermore, MR cannot account for the potential existence of non-linear associations. Therefore, it is our opinion that it would be prudent to avoid deficiency in these micronutrients to mitigate severe to fatal COVID-19 and that this hypothesis warrants more detailed study. Thus, more extensive epidemiological studies are required to investigate the possible role of micronutrients in COVID-19 severity, as the MR results presented here cannot be considered definitive.

## Data Availability Statement

The original contributions presented in the study are included in the article/Supplementary Material, further inquiries can be directed to the corresponding authors.

## Ethics Statement

Ethical review and approval was not required for the study on human participants in accordance with the local legislation and institutional requirements. The patients/participants provided their written informed consent to participate in this study.

## Author Contributions

DH and KT conceived the study, supervised the work of ND and EB, and reviewed and approved the submitted version. ND and DH drafted the manuscript. KT and EB performed the analysis and wrote the statistics section of the manuscript. All authors revised and approved the manuscript for publication.

## Funding

ND was funded by a UCD College of Science Ad Astra PhD scholarship (R20443; supervisor, DH).

## Conflict of Interest

The authors declare that the research was conducted in the absence of any commercial or financial relationships that could be construed as a potential conflict of interest.

## Publisher's Note

All claims expressed in this article are solely those of the authors and do not necessarily represent those of their affiliated organizations, or those of the publisher, the editors and the reviewers. Any product that may be evaluated in this article, or claim that may be made by its manufacturer, is not guaranteed or endorsed by the publisher.

## Footnotes

^1^ https://www.covid19hg.org/results/

^2^http://www.phenoscanner.medschl.cam.ac.uk/ (69).

## References

[1] WHO Coronavirus Disease (COVID-19) Dashboard. Available online at: https://covid19.who.int/ (accessed December23, 2021).

[2] Bhaskar S Sinha A Banach M Mittoo S Weissert R Kass JS. Cytokine storm in COVID-19-immunopathological mechanisms, clinical considerations, and therapeutic approaches: the REPROGRAM consortium position paper. Front Immunol. (2020) 11:1648. 10.3389/fimmu.2020.01648 32754159 PMC7365905

[3] Figliozzi S Masci PG Ahmadi N Tondi L Koutli E Aimo A. Predictors of adverse prognosis in COVID-19: a systematic review and meta-analysis. Eur J Clin Invest. (2020) 50:e13362. 10.1111/eci.13362 32726868

[4] Soeroto AY Soetedjo NN Purwiga A Santoso P Kulsum ID Suryadinata H. Effect of increased BMI and obesity on the outcome of COVID-19 adult patients: a systematic review and meta-analysis. Diabetes Metab Syndr. (2020) 14:1897–904. 10.1016/j.dsx.2020.09.029 33007661 PMC7521380

[5] Sallis R Young DR Tartof SY Sallis JF Sall J Li Q. Physical inactivity is associated with a higher risk for severe COVID-19 outcomes: a study in 48 440 adult patients. Br J Sports Med. (2021). 10.2139/ssrn.3770671 33849909

[6] Cheng S Zhao Y Wang F Chen Y Kaminga AC Xu H. Comorbidities' potential impacts on severe and non-severe patients with COVID-19: a systematic review and meta-analysis. Medicine. (2021) 100:e24971. 10.1097/MD.0000000000024971 33761654 PMC9281964

[7] European Medicines Agency Website. Available online at: https://www.ema.europa.eu/en (accessed June 71, 2021).

[8] Gombart AF Pierre A Maggini S. A review of micronutrients and the immune system-working in harmony to reduce the risk of infection. Nutrients. (2020) 12:236. 10.3390/nu12010236 31963293 PMC7019735

[9] Maggini S Pierre A Calder PC. Immune function and micronutrient requirements change over the life course. Nutrients. (2018) 10:1531. 10.3390/nu10101531 30336639 PMC6212925

[10] Mansur JL Tajer C Mariani J Inserra F Ferder L Manucha W. Vitamin D high doses supplementation could represent a promising alternative to prevent or treat COVID-19 infection. Clin Investig Arterioscler. (2020) 32:267–77. 10.1016/j.artere.2020.11.003 32718670 PMC7256522

[11] Moghaddam A Heller RA Sun Q Seelig J Cherkezov A Seibert L. Selenium deficiency is associated with mortality risk from COVID-19. Nutrients. (2020) 12:2098. 10.3390/nu12072098 32708526 PMC7400921

[12] Jothimani D Kailasam E Danielraj S Nallathambi B Ramachandran H Sekar P. COVID-19: poor outcomes in patients with zinc deficiency. Int J Infect Dis. (2020) 100:343–9. 10.1016/j.ijid.2020.09.014 32920234 PMC7482607

[13] Davies NM Holmes MV Davey Smith G. Reading mendelian randomisation studies: a guide, glossary, and checklist for clinicians. BMJ. (2018) 362:k601. 10.1136/bmj.k601 30002074 PMC6041728

[14] Kotur N Skakic A Klaassen K Gasic V Zukic B Skodric-Trifunovic V. Association of Vitamin D, Zinc and Selenium related genetic variants with COVID-19 disease severity. Front Nutr. (2021) 8:689419. 10.3389/fnut.2021.689419 34150833 PMC8211741

[15] Razdan K Singh K Singh D. Vitamin D levels and COVID-19 susceptibility: is there any correlation? Med Drug Discov. (2020) 7:100051. 10.1016/j.medidd.2020.100051 32835212 PMC7266578

[16] Li X van Geffen J van Weele M Zhang X He Y Meng X. An observational and Mendelian randomisation study on vitamin D and COVID-19 risk in UK Biobank. Sci Rep. (2021) 11:18262. 10.1038/s41598-021-97679-5 34521884 PMC8440633

[17] Amin HA Drenos F. No evidence that vitamin D is able to prevent or affect the severity of COVID-19 in individuals with European ancestry: a mendelian randomisation study of open data. BMJ Nutr Prev Health. (2021) 4:42–8. 10.1136/bmjnph-2020-000151 34308111 PMC7798425

[18] Cui Z Tian Y. Using genetic variants to evaluate the causal effect of serum vitamin D concentration on COVID-19 susceptibility, severity and hospitalization traits: a Mendelian randomization study. J Transl Med. (2021) 19:300. 10.1186/s12967-021-02973-5 34246301 PMC8271325

[19] Butler-Laporte G Nakanishi T Mooser V Morrison DR Abdullah T Adeleye O. Vitamin D and COVID-19 susceptibility and severity in the COVID-19 host genetics initiative: a mendelian randomization study. PLoS Med. (2021) 18:e1003605. 10.1371/journal.pmed.1003605 34061844 PMC8168855

[20] Kelly A Levine MA. Hypocalcemia in the critically ill patient. J Intensive Care Med. (2013) 28:166–77. 10.1177/0885066611411543 21841146

[21] Trebak M Kinet JP. Calcium signalling in T cells. Nat Rev Immunol. (2019) 19:154–69. 10.1038/s41577-018-0110-7 30622345 PMC6788797

[22] Sun JK Zhang WH Zou L Liu Y Li JJ Kan XH. Serum calcium as a biomarker of clinical severity and prognosis in patients with coronavirus disease 2019. Aging. (2020) 12:11287–95. 10.18632/aging.103526 32589164 PMC7343468

[23] Bennouar S Cherif AB Kessira A Bennouar DE Abdi S. Vitamin D deficiency and low serum calcium as predictors of poor prognosis in patients with severe COVID-19. J Am Coll Nutr. (2021) 40:104–10. 10.1080/07315724.2020.1856013 33434117 PMC7814570

[24] Galmes S Serra F Palou A. Current state of evidence: influence of nutritional and nutrigenetic factors on immunity in the COVID-19 pandemic framework. Nutrients. (2020) 12:2738. 10.3390/nu12092738 32911778 PMC7551697

[25] Percival SS. Copper and immunity. Am J Clin Nutr. (1998) 67(5 Suppl):1064S−8S. 10.1093/ajcn/67.5.1064S 9587153

[26] Johnson MA Fischer JG Kays SE. Is copper an antioxidant nutrient? Crit Rev Food Sci Nutr. (1992) 32:1–31. 10.1080/10408399209527578 1290583

[27] Besold AN Culbertson EM Culotta VC. The Yin and Yang of copper during infection. J Biol Inorg Chem. (2016) 21:137–44. 10.1007/s00775-016-1335-1 26790881 PMC5535265

[28] Dhur A Galan P Hercberg S. Folate status and the immune system. Prog Food Nutr Sci. (1991) 15:43–60. 1887065

[29] Abe I Shirato K Hashizume Y Mitsuhashi R Kobayashi A Shiono C. Folate-deficiency induced cell-specific changes in the distribution of lymphocytes and granulocytes in rats. Environ Health Prev Med. (2013) 18:78–84. 10.1007/s12199-012-0286-6 22644659 PMC3541812

[30] Courtemanche C Elson-Schwab I Mashiyama ST Kerry N Ames BN. Folate deficiency inhibits the proliferation of primary human CD8+ T lymphocytes *in vitro*. J Immunol. (2004) 173:3186–92. 10.4049/jimmunol.173.5.3186 15322179

[31] Duthie SJ Horgan G de Roos B Rucklidge G Reid M Duncan G. Blood folate status and expression of proteins involved in immune function, inflammation, and coagulation: biochemical and proteomic changes in the plasma of humans in response to long-term synthetic folic acid supplementation. J Proteome Res. (2010) 9:1941–50. 10.1021/pr901103n 20143872

[32] Agoro R Taleb M Quesniaux VFJ Mura C. Cell iron status influences macrophage polarization. PLoS ONE. (2018) 13:e0196921. 10.1371/journal.pone.0196921 29771935 PMC5957380

[33] Huang Z Rose AH Hoffmann PR. The role of selenium in inflammation and immunity: from molecular mechanisms to therapeutic opportunities. Antioxid Redox Signal. (2012) 16:705–43. 10.1089/ars.2011.4145 21955027 PMC3277928

[34] Khatiwada S Subedi A. A mechanistic link between selenium and coronavirus disease 2019 (COVID-19). Curr Nutr Rep. (2021) 10:125–36. 10.1007/s13668-021-00354-4 33835432 PMC8033553

[35] Maares M Haase H. Zinc and immunity: an essential interrelation. Arch Biochem Biophys. (2016) 611:58–65. 10.1016/j.abb.2016.03.022 27021581

[36] Sivaprasad S Chong NV. The complement system and age-related macular degeneration. Eye. (2006) 20:867–72. 10.1038/sj.eye.6702176 16410816

[37] Read SA Obeid S Ahlenstiel C Ahlenstiel G. The role of zinc in antiviral immunity. Adv Nutr. (2019) 10:696–710. 10.1093/advances/nmz013 31305906 PMC6628855

[38] Pal A Squitti R Picozza M Pawar A Rongioletti M Dutta AK. Zinc and COVID-19: basis of current clinical trials. Biol Trace Elem Res. (2020). 10.1007/s12011-020-02437-9 PMC7580816 33094446

[39] Yang C Ma X Wu J Han J Zheng Z Duan H. Low serum calcium and phosphorus and their clinical performance in detecting COVID-19 patients. J Med Virol. (2021) 93:1639–51. 10.1002/jmv.26515 32926424

[40] Wallace TC. Combating COVID-19 and building immune resilience: a potential role for magnesium nutrition? J Am Coll Nutr. (2020) 39:685–93. 10.1080/07315724.2020.1785971 32649272

[41] Tang CF Ding H Jiao RQ Wu XX Kong LD. Possibility of magnesium supplementation for supportive treatment in patients with COVID-19. Eur J Pharmacol. (2020) 886:173546. 10.1016/j.ejphar.2020.173546 32931782 PMC7486870

[42] Qian B Shen S Zhang J Jing P. Effects of Vitamin B6 deficiency on the composition and functional potential of T cell populations. J Immunol Res. (2017) 2017:2197975. 10.1155/2017/2197975 28367454 PMC5358464

[43] Ha C Miller LT Kerkvliet NI. The effect of vitamin B6 deficiency on cytotoxic immune responses of T cells, antibodies, and natural killer cells, and phagocytosis by macrophages. Cell Immunol. (1984) 85:318–29. 10.1016/0008-8749(84)90246-6 6608998

[44] Tamura J Kubota K Murakami H Sawamura M Matsushima T Tamura T. Immunomodulation by vitamin B12: augmentation of CD8+ T lymphocytes and natural killer (NK) cell activity in vitamin B12-deficient patients by methyl-B12 treatment. Clin Exp Immunol. (1999) 116:28–32. 10.1046/j.1365-2249.1999.00870.x 10209501 PMC1905232

[45] Zhao LG Zhang QL Zheng JL Li HL Zhang W Tang WG. Dietary, circulating beta-carotene and risk of all-cause mortality: a meta-analysis from prospective studies. Sci Rep. (2016) 6:26983. 10.1038/srep26983 27243945 PMC4886629

[46] Willcox JK Ash SL Catignani GL. Antioxidants and prevention of chronic disease. Crit Rev Food Sci Nutr. (2004) 44:275–95. 10.1080/10408690490468489 15462130

[47] Huang Z Liu Y Qi G Brand D Zheng SG. Role of Vitamin A in the Immune System. J Clin Med. (2018) 7:258. 10.3390/jcm7090258 30200565 PMC6162863

[48] Heller RA Sun Q Hackler J Seelig J Seibert L Cherkezov A. Prediction of survival odds in COVID-19 by zinc, age and selenoprotein P as composite biomarker. Redox Biol. (2021) 38:101764. 10.1016/j.redox.2020.101764 33126054 PMC7574778

[49] Tsilidis KK Papadimitriou N Dimou N Gill D Lewis SJ Martin RM. Genetically predicted circulating concentrations of micronutrients and risk of colorectal cancer among individuals of European descent: a Mendelian randomization study. Am J Clin Nutr. (2021) 113:1490–502. 10.1093/ajcn/nqab003 33740060 PMC8168352

[50] Benyamin B Esko T Ried JS Radhakrishnan A Vermeulen SH Traglia M. Novel loci affecting iron homeostasis and their effects in individuals at risk for hemochromatosis. Nature commun. (2014) 5:4926. 10.1038/ncomms5926 25352340 PMC4215164

[51] Cornelis MC Fornage M Foy M Xun P Gladyshev VN Morris S. Genome-wide association study of selenium concentrations. Hum Mol Genet. (2015) 24:1469–77. 10.1093/hmg/ddu546 25343990 PMC4321444

[52] Evans DM Zhu G Dy V Heath AC Madden PA Kemp JP. Genome-wide association study identifies loci affecting blood copper, selenium and zinc. Hum Mol Genet. (2013) 22:3998–4006. 10.1093/hmg/ddt239 23720494 PMC3766178

[53] Ferrucci L Perry JR Matteini A Perola M Tanaka T Silander K. Common variation in the beta-carotene 15,15'-monooxygenase 1 gene affects circulating levels of carotenoids: a genome-wide association study. Am J Hum Genet. (2009) 84:123–33. 10.1016/j.ajhg.2008.12.019 19185284 PMC2668002

[54] Grarup N Sulem P Sandholt CH Thorleifsson G Ahluwalia TS Steinthorsdottir V. Genetic architecture of vitamin B12 and folate levels uncovered applying deeply sequenced large datasets. PLoS Genet. (2013) 9:e1003530. 10.1371/journal.pgen.1003530 23754956 PMC3674994

[55] Hazra A Kraft P Lazarus R Chen C Chanock SJ Jacques P. Genome-wide significant predictors of metabolites in the one-carbon metabolism pathway. Hum Mol Genet. (2009) 18:4677–87. 10.1093/hmg/ddp428 19744961 PMC2773275

[56] Kestenbaum B Glazer NL Köttgen A Felix JF Hwang SJ Liu Y. Common genetic variants associate with serum phosphorus concentration. J Am Soc Nephrol. (2010) 21:1223–32. 10.1681/ASN.2009111104 20558539 PMC3152230

[57] Meyer TE Verwoert GC Hwang SJ Glazer NL Smith AV van Rooij FJ. Genome-wide association studies of serum magnesium, potassium, and sodium concentrations identify six Loci influencing serum magnesium levels. PLoS Genet. (2010) 6:e1001045. 10.1371/journal.pgen.1001045 20700443 PMC2916845

[58] Ong J-S Dixon-Suen SC Han X An J Esophageal Cancer C Me Research T. A comprehensive re-assessment of the association between vitamin D and cancer susceptibility using Mendelian randomization. Nat Commun. (2021) 12:246. 10.1038/s41467-020-20368-w 33431812 PMC7801600

[59] O'Seaghdha CM Wu H Yang Q Kapur K Guessous I Zuber AM. Meta-analysis of genome-wide association studies identifies six new Loci for serum calcium concentrations. PLoS Genet. (2013) 9:e1003796. 10.1371/journal.pgen.1003796 24068962 PMC3778004

[60] Sinnott-Armstrong N Tanigawa Y Amar D Mars N Benner C Aguirre M. Genetics of 35 blood and urine biomarkers in the UK Biobank. Nat Genet. (2021) 53:185–94. 10.1038/s41588-020-00757-z 33462484 PMC7867639

[61] Brion MJ Shakhbazov K Visscher PM. Calculating statistical power in Mendelian randomization studies. Int J Epidemiol. (2013) 42:1497–501. 10.1093/ije/dyt179 24159078 PMC3807619

[62] Initiative C-HG. Mapping the human genetic architecture of COVID-19. Nature. (2021) 600:472–7. 10.1038/s41586-021-03767-x 34237774 PMC8674144

[63] Lawlor DA Harbord RM Sterne JA Timpson N Davey Smith G. Mendelian randomization: using genes as instruments for making causal inferences in epidemiology. Stat Med. (2008) 27:1133–63. 10.1002/sim.3034 17886233

[64] Burgess S Butterworth A Thompson SG. Mendelian randomization analysis with multiple genetic variants using summarized data. Genet Epidemiol. (2013) 37:658–65. 10.1002/gepi.21758 24114802 PMC4377079

[65] Rodriguez-Barranco M Tobias A Redondo D Molina-Portillo E Sanchez MJ. Standardizing effect size from linear regression models with log-transformed variables for meta-analysis. BMC Med Res Methodol. (2017) 17:44. 10.1186/s12874-017-0322-8 28302052 PMC5356327

[66] Haycock PC Burgess S Wade KH Bowden J Relton C Davey Smith G. Best (but oft-forgotten) practices: the design, analysis, and interpretation of Mendelian randomization studies. Am J Clin Nutr. (2016) 103:965–78. 10.3945/ajcn.115.118216 26961927 PMC4807699

[67] Burgess S Thompson SG Collaboration CCG. Avoiding bias from weak instruments in Mendelian randomization studies. Int J Epidemiol. (2011) 40:755–64. 10.1093/ije/dyr036 21414999

[68] Greco MF Minelli C Sheehan NA Thompson JR. Detecting pleiotropy in Mendelian randomisation studies with summary data and a continuous outcome. Stat Med. (2015) 34:2926–40. 10.1002/sim.6522 25950993

[69] Staley JR Blackshaw J Kamat MA Ellis S Surendran P Sun BB. PhenoScanner: a database of human genotype–phenotype associations. Bioinformatics. (2016) 32:3207–9. 10.1093/bioinformatics/btw373 27318201 PMC5048068

[70] Bowden J Davey Smith G Burgess S. Mendelian randomization with invalid instruments: effect estimation and bias detection through Egger regression. Int J Epidemiol. (2015) 44:512–25. 10.1093/ije/dyv080 26050253 PMC4469799

[71] Bowden J Davey Smith G Haycock PC Burgess S. Consistent Estimation in mendelian randomization with some invalid instruments using a weighted median estimator. Genet Epidemiol. (2016) 40:304–14. 10.1002/gepi.21965 27061298 PMC4849733

[72] Burgess S Foley CN Allara E Staley JR Howson JMM. A robust and efficient method for Mendelian randomization with hundreds of genetic variants. Nat Commun. (2020) 11:376. 10.1038/s41467-019-14156-4 31953392 PMC6969055

[73] Verbanck M Chen CY Neale B Do R. Detection of widespread horizontal pleiotropy in causal relationships inferred from Mendelian randomization between complex traits and diseases. Nat Genet. (2018) 50:693–8. 10.1038/s41588-018-0099-7 29686387 PMC6083837

[74] Teng MS Wu S Hsu LA Chou HH Ko YL. Pleiotropic Effects of Functional MUC1 Variants on Cardiometabolic, Renal, and Hematological Traits in the Taiwanese Population. Int J Mol Sci. (2021) 22:10641. 10.3390/ijms221910641 34638981 PMC8509060

[75] Li F-Y Chaigne-Delalande B Kanellopoulou C Davis JC Matthews HF Douek DC. Second messenger role for Mg2+ revealed by human T-cell immunodeficiency. Nature. (2011) 475:471–6. 10.1038/nature10246 21796205 PMC3159560

[76] Juan R Otim I Nabalende H Legason ID Reynolds SJ Ogwang MD. Plasma magnesium is inversely associated with Epstein-Barr virus load in peripheral blood and Burkitt lymphoma in Uganda. Cancer Epidemiol. (2018) 52:70–4. 10.1016/j.canep.2017.12.004 29248801 PMC5785547

[77] Chaigne-Delalande B Li F-Y O'Connor GM Lukacs MJ Jiang P Zheng L. Mg^2^+regulates cytotoxic functions of NK and CD8 T cells in chronic EBV infection through NKG2D. Science. (2013) 341:186–91. 10.1126/science.1240094 23846901 PMC3894782

[78] Diao B Huang X Guo S Yang C Liu G Chen Y. MAGT1-mediated disturbance of Mg2+ homeostasis lead to exhausted of HBV-infected NK and CD8+ T cells. Sci Rep. (2017) 7:13594. 10.1038/s41598-017-11522-4 29051561 PMC5648775

[79] Varchetta S Mele D Oliviero B Mantovani S Ludovisi S Cerino A. Unique immunological profile in patients with COVID-19. Cell Mol Immunol. (2021) 18:604–12. 10.1038/s41423-020-00557-9 33060840 PMC7557230

[80] Kumrungsee T Zhang P Chartkul M Yanaka N Kato N. Potential Role of Vitamin B6 in Ameliorating the Severity of COVID-19 and Its Complications. Front Nutr. (2020) 7:562051. 10.3389/fnut.2020.562051 33195363 PMC7658555

[81] Hackler J Heller RA Sun Q Schwarzer M Diegmann J Bachmann M. Relation of serum copper status to survival in COVID-19. Nutrients. (2021) 13:1898. 10.3390/nu13061898 34072977 PMC8229409

[82] Di Filippo L Formenti AM Rovere-Querini P Carlucci M Conte C Ciceri F. Hypocalcemia is highly prevalent and predicts hospitalization in patients with COVID-19. Endocrine. (2020) 68:475–8. 10.1007/s12020-020-02383-5 32533508 PMC7292572

[83] Zhou X Chen D Wang L Zhao Y Wei L Chen Z. Low serum calcium: a new, important indicator of COVID-19 patients from mild/moderate to severe/critical. Biosci Rep. (2020) 40:BSR20202690. 10.1042/BSR20202690 33252122 PMC7755121

[84] Cappellini F Brivio R Casati M Cavallero A Contro E Brambilla P. Low levels of total and ionized calcium in blood of COVID-19 patients. Clin Chem Lab Med. (2020) 58:e171–3. 10.1515/cclm-2020-0611 32459190

[85] Acosta-Elias J Espinosa-Tanguma R. The Folate concentration and/or folic acid metabolites in plasma as factor for COVID-19 infection. Front Pharmacol. (2020) 11:1062. 10.3389/fphar.2020.01062 32765270 PMC7379025

[86] Im JH Je YS Baek J Chung MH Kwon HY Lee JS. Nutritional status of patients with COVID-19. Int J Infect Dis. (2020) 100:390–3. 10.1016/j.ijid.2020.08.018 32795605 PMC7418699

[87] Alexander J Tinkov A Strand TA Alehagen U Skalny A Aaseth J. Early nutritional interventions with zinc, selenium and vitamin D for raising anti-viral resistance against progressive COVID-19. Nutrients. (2020) 12:2358. 10.3390/nu12082358 32784601 PMC7468884

[88] Sobczyk MK Gaunt TR. The effect of circulating zinc, selenium, copper and vitamin K1 on COVID-19 outcomes: a mendelian randomization study. Nutrients. (2022) 14:233. 10.3390/nu14020233 35057415 PMC8780111

[89] Zhao K Huang J Dai D Feng Y Liu L Nie S. Serum iron level as a potential predictor of coronavirus disease 2019 severity and mortality: a retrospective study. Open Forum Infect Dis. (2020) 7:ofaa250. 10.1093/ofid/ofaa250 32661499 PMC7337740

[90] Perricone C Bartoloni E Bursi R Cafaro G Guidelli GM Shoenfeld Y. COVID-19 as part of the hyperferritinemic syndromes: the role of iron depletion therapy. Immunol Res. (2020) 68:213–24. 10.1007/s12026-020-09145-5 32681497 PMC7366458

[91] Naffaa ME Mustafa M Azzam M Nasser R Andria N Azzam ZS. Serum inorganic phosphorus levels predict 30-day mortality in patients with community acquired pneumonia. BMC Infect Dis. (2015) 15:332. 10.1186/s12879-015-1094-6 26268323 PMC4535260

[92] Fiedor J Burda K. Potential role of carotenoids as antioxidants in human health and disease. Nutrients. (2014) 6:466–88. 10.3390/nu6020466 24473231 PMC3942711

[93] Grune T Lietz G Palou A Ross AC Stahl W Tang G. Beta-carotene is an important vitamin A source for humans. J Nutr. (2010) 140:2268S−85. 10.3945/jn.109.119024 PMC3139236 20980645

[94] Cook NR Albert CM Gaziano JM Zaharris E MacFadyen J Danielson E. A randomized factorial trial of vitamins C and E and beta carotene in the secondary prevention of cardiovascular events in women: results from the women's antioxidant cardiovascular study. Arch Intern Med. (2007) 167:1610–8. 10.1001/archinte.167.15.1610 17698683 PMC2034519

[95] Goodman GE Thornquist MD Balmes J Cullen MR Meyskens FL Jr Omenn GS. The beta-carotene and retinol efficacy trial: incidence of lung cancer and cardiovascular disease mortality during 6-Year follow-up after stopping beta-carotene and retinol supplements. J Natl Cancer Inst. (2004) 96:1743–50. 10.1093/jnci/djh320 15572756

[96] Foo YZ Rhodes G Simmons LW. The carotenoid beta-carotene enhances facial color, attractiveness and perceived health, but not actual health, in humans. Behavioral Ecology. (2017) 28:570–8. 10.1093/beheco/arw188

[97] Hughes DA. Dietary carotenoids and human immune function. Nutrition. (2001) 17:823–7. 10.1016/S0899-9007(01)00638-4 11684388

[98] Batista KS Cintra VM Lucena PAF Manhães-de-Castro R Toscano AE Costa LP. The role of vitamin B12 in viral infections: a comprehensive review of its relationship with the muscle–gut–brain axis and implications for SARS-CoV-2 infection. Nutr Rev. (2022) 80:561–78. 10.1093/nutrit/nuab092 34791425 PMC8689946

[99] Shakeri H Azimian A Ghasemzadeh-Moghaddam H Safdari M Haresabadi M Daneshmand T. Evaluation of the relationship between serum levels of zinc, vitamin B12, vitamin D, and clinical outcomes in patients with COVID-19. J Med Virol. (2022) 94:141–6. 10.1002/jmv.27277 34406674 PMC8426973

